# Diagnosis pathway for patients with amyotrophic lateral sclerosis: retrospective analysis of the US Medicare longitudinal claims database

**DOI:** 10.1186/1471-2377-13-160

**Published:** 2013-11-04

**Authors:** James R Williams, David Fitzhenry, Lauren Grant, Derek Martyn, Douglas A Kerr

**Affiliations:** 1 Biogen Idec, 14 Cambridge Center, Cambridge, MA 02142, USA; 2 Trinity Partners, LLC, 230 3rd Ave, Waltham, MA, USA

**Keywords:** Amyotrophic lateral sclerosis, Pre-diagnosis, Diagnostic delay, Diagnostic tests, Symptoms

## Abstract

**Background:**

Initial symptoms of amyotrophic lateral sclerosis (ALS) are often subtle and can delay diagnosis. This exploratory analysis was conducted to better characterize the pre-diagnosis pathway undertaken by patients with ALS in the US Centers for Medicare & Medicaid Services Medicare longitudinal claims database.

**Methods:**

Quarterly Medicare claims data were analyzed to determine the pre-diagnosis pathway for an ALS patient cohort that included patients aged ≥ 65 years with ≥ 2 ALS claims (International Classification of Diseases, Ninth Revision, Clinical Modification code 335.20) between the first quarter of 2007 and the fourth quarter of 2009, and were enrolled in Medicare ≥ 2 years before the first ALS claim (diagnosis). A cohort of Medicare patients without claims for motor neuron diseases were identified for comparison. A subset of these patients with ≥ 3 years of claims data was included in a time to diagnosis analysis. Data extraction included the most common initial symptoms of ALS, the time from first ALS symptom to diagnosis, and the diagnostic procedures performed before the diagnosis of ALS.

**Results:**

A total of 399 patients met the inclusion criteria and were included in the ALS cohort; 272 patients were included in the time to diagnosis cohort. Before the quarter of diagnosis, symptoms that were more frequently seen in the ALS cohort than the general Medicare cohort included muscle weakness, lack of coordination and speech/swallowing difficulties. Limb-onset ALS (74%) was more common than bulbar-onset ALS (17%). Median time to diagnosis for limb- and bulbar-onset patients was 2.5 years and 1.25 years, respectively. The most common tests conducted before the quarter of diagnosis included sensory and motor nerve conduction tests, imaging studies, and electromyography; however, a substantial number of patients did not receive any nerve conduction testing. Motor nerve conduction testing in patients with bulbar-onset ALS had the largest impact on time to diagnosis.

**Conclusions:**

This analysis describes a diagnostic delay for patients with ALS in the US Medicare population, similar to previous reports. The development of tools and ongoing education that can help to identify patients with ALS earlier in their disease course is needed.

## Background

Amyotrophic lateral sclerosis (ALS) is a progressive neurodegenerative disease affecting the upper and lower motor neurons [1]. ALS is fatal; death generally occurs within 2 years after symptom onset although up to 10% of patients with ALS survive more than 10 years [2]. Prognostic factors have been identified for ALS: 1) advanced age is associated with increased incidence of ALS; 2) women have a shorter survival time than men; and 3) bulbar-onset ALS is associated with shorter survival [2]. Currently, there is no cure for ALS and treatment is largely supportive. Riluzole is the only drug approved by the US Food and Drug Administration for the pharmacologic treatment of ALS; however, the effect of riluzole on survival is modest [3].

The initial symptoms of ALS are often subtle (limb or shoulder weakness, difficulty walking) despite the deadly nature of the disease [1]. Unrecognized symptoms can result in delays in the diagnosis of ALS as well as misdiagnosis. Retrospective reviews have demonstrated a delay from symptom onset to diagnosis that has remained unchanged for more than a decade and ranges from 8.0 to 15.6 months [4]. Diagnostic delay may deter timely beneficial intervention, heighten psychological distress, prevent clinical trial participation, and add to financial and social support issues [5]. In addition, delays in diagnosis also may prevent timely referral to centers offering a multidisciplinary approach to ALS care, which has been shown to prolong survival [6,7]. Patients receiving care from a multidisciplinary team also have reported a higher quality of life [8]. Misdiagnosis has been reported to result in unnecessary interventions, such as surgery [9-11]. Attempts to improve the diagnostic delay in ALS have identified reasons for the delay including atypical symptoms of ALS, referrals to other departments before referral to a neurologist, and a lack of recognition of symptoms of the ALS in the general practitioner office [12].

This exploratory analysis was undertaken to investigate the initial symptoms and time to diagnosis for patients with ALS in the US Centers for Medicare & Medicaid Services (CMS) longitudinal claims dataset. Key diagnostic tests prior to the quarter of diagnosis also were examined. This was a pilot analysis for a broader project meant to identify “Red Flag” signs and symptoms that could be used to encourage expedited referrals in patients with early ALS symptoms.

## Methods

### Data source

This retrospective study used data from the CMS longitudinal claims dataset (http://www.resdac.org). In the United States, Medicare provides coverage to those aged 65 years and older and offers coverage for those who are younger than 65 years with a disability, such as patients with ALS [13]. The Medicare dataset used in this study is made available by the US Department of Health and Human Services as a way to improve transparency and provide a source of information that can be used to improve health care [14]. Included in the dataset were adjudicated claims data for approximately 80% of Medicare recipients covered by traditional fee-for-service plans (i.e., patients with Medicare Advantage plans were not included); the data set is maintained by the CMS. All (100%) patient institutional claims between the first quarter (Q1) of 2005 and the fourth quarter (Q4) of 2009 were included in the dataset. In addition, physician office data were available for 5% of patients in the database. These patients were identified for inclusion in the 5% physician office sample using the last 2 digits of their Medicare account number. Once included, beneficiaries remain in the physician office sample from enrollment until death. In the public datasets, data were classified only by quarter of service; dates of service were not available.

### Study cohort descriptions

Claims data from Q1 2005 to Q4 2009 were reviewed to identify patients who met the study inclusion criteria. Patients were included in the ALS cohort if the following criteria were met: patients had to be aged 65 years or older, had a first ALS claim (International Classification of Diseases, Ninth Revision, Clinical Modification [ICD-9-CM] code 335.20) and two or more ALS claims (ICD-9-CM code 335.20) from Q1 2007 to Q4 2009, and were in the CMS dataset for 2 years or more before the first ALS claim, with available physician office data. To ensure there was a minimum of 3 years of prior claims to examine for ALS symptoms present prior to diagnosis, the time to diagnosis analysis was based on a cohort of 272 patients with an initial ALS claim between Q1 2008 and Q4 2009. In addition, data from the general Medicare population (Q1 2008 to Q4 2009) formed a Medicare cohort for comparison.

### Study design

Adjudicated inpatient, outpatient, and physician office CMS Medicare claims were reviewed for this analysis. Data extraction included the most common initial symptoms of ALS, time from first ALS symptom to diagnosis, and diagnostic procedures performed before the diagnosis of ALS. Symptom, procedure, and diagnostic codes were evaluated in patients in the ALS cohort; symptom and diagnostic codes were evaluated in the Medicare cohort.

Symptoms of ALS were classified based on the likelihood that they were related to a patient’s disease. A multi-step process was used to develop a classification of likelihood (high or moderate) that the symptoms reported in the medical claim were ALS related. Following an initial review of available literature, a patient record analysis was performed on the complete claims history of 50 Medicare patients with a diagnosis of ALS to review the frequency of reported symptoms before diagnosis. Based on this analysis, an initial list of possible ALS-related symptoms was generated and reviewed by one of the authors (DK) who has significant clinical experience in the diagnosis and treatment of ALS. Symptoms on the list were then further classified as high or moderate likelihood symptoms and were used to determine a patient’s initial ALS symptom (Table 1).

**Table 1:** Codes identified as high or moderate likelihood of being first ALS symptom

	**Priority**	**Subgroup**	**Diagnosis of interest**	**ICD-9-CM codes**
Bulbar	High	Speech	Developmental speech, language disorder	315.39
			Speech and language deficits (including aphasia, dysphasia, voice resonance disorder, speech disturbance, dysarthria)	438.1X, 784.3, 784.4X, 784.5X
			Dysphagia	787.2, 438.82
		Swallowing	Disturbance, salivary secretion	527.7
			Salivary gland disorder	527.8
			Pain, throat	784.1
Limb	High	Muscle strength	Atrophy, muscular disuse	728.2
			Muscle weakness (generalized)	728.87
		Gait	Difficulty walking	719.7
			Gait abnormality	781.2
			Lack of coordination	781.3
		Involuntary muscle movement	Cramping	729.82
			Spasm	728.85
			Twitching/fasciculation	781.0
		Myopathy	Myopathy (including myopathy with weakness)	359.X
	Moderate	Pain	Pain in joint	719.4
			Pain in limb	729.5
		Other	Disorder of muscle and/or ligament	728.X

ALS, amyotrophic lateral sclerosis; ICD-9-CM, International Classification of Diseases, Ninth Revision, Clinical Modification.

Symptoms defined in this study as “high likelihood” included symptoms that were highly associated with ALS based on a literature review of ALS symptomatology, frequency in the 50-patient Medicare cohort, and confirmation by the ALS specialist. Because of the close association, a code for a single high likelihood symptom was considered an indication of the first ALS symptom. Symptoms defined as “moderate likelihood” included those associated with ALS based on frequency in the 50-patient Medicare cohort and confirmed by the ALS specialist. Coding for two moderate likelihood symptoms in a single quarter was required to define the first ALS symptoms. The first instance of either a single high likelihood symptom or two moderate likelihood symptoms was used to define the quarter of first ALS symptom. Symptoms were further grouped into bulbar, limb, nerve, respiratory, and other symptom categories to reflect the common symptom classes present in ALS.

The prevalence of diagnostic codes present in medical claims 2 years prior to the first ALS claim was tabulated. Multiple diagnostic codes related to a single sign or symptom were grouped together. A prevalence rate ratio was calculated for identified symptom or diagnostic codes by dividing the percentage of patients in the ALS cohort having a given symptom by the percentage of patients in the Medicare cohort with the same symptom. Data for the signs and symptoms with a prevalence of at least 10% in the ALS cohort or with a prevalence ratio of at least 5 were identified. Data also were reviewed to identify a time from first symptom(s) to diagnosis of ALS and the frequency of use of common diagnostic procedures. The analyses were exploratory in nature. No hypothesis testing was conducted.

## Results

**Figure 1:**
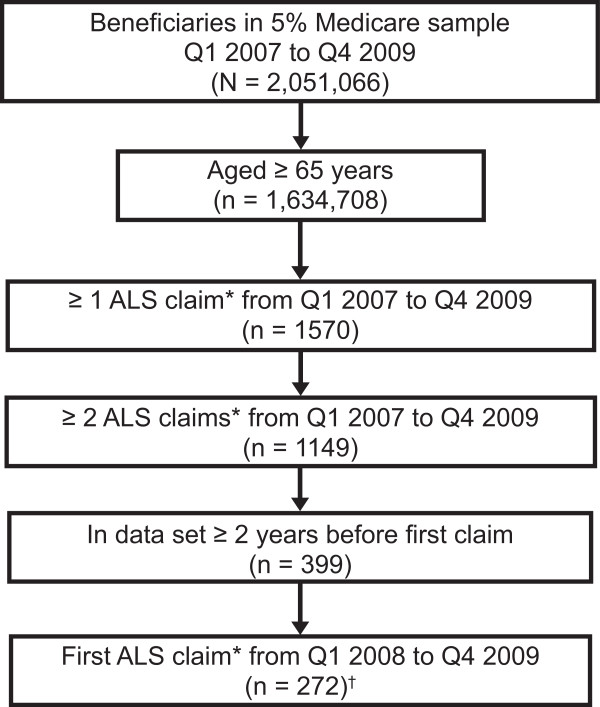
**Amyotrophic lateral sclerosis (ALS) cohort eligibility.** *International Classification of Diseases, Ninth Revision, Clinical Modification code 335.20. ^†^Population for time to diagnosis evaluation. Q1, quarter one; Q4, quarter four.

The Medicare 5% sample housed information for just over 2 million patients with physician office data during the study period. A total of 399 patients (53% female) in the database met the inclusion criteria (Figure 1). The percentage of females (55%) in the Medicare 5% sample over the timeframe of interest (2005–2009) also was slightly higher than males. Within our defined ALS cohort, 272 qualified for the evaluation of time to diagnosis based on their first ALS claim occurring between Q1 2008 and Q4 2009.

In the time to diagnosis cohort, limb-onset ALS (74%) was more common than bulbar-onset ALS (17%); evidence of both limb and bulbar symptoms occurring concurrently at onset was noted in 8% of patients. Median time to diagnosis from first high and/or moderate likelihood limb or bulbar symptom in the 272 patient cohort was 2.25 years. The median time to diagnosis was shorter for patients with bulbar-onset than for patients with limb-onset (1.25 years vs 2.5 years, respectively), and, in the subgroup where limb and bulbar symptoms appeared at the same time, the median time to diagnosis was only 0.25 years. In the bulbar-onset subgroup, 38% of patients were diagnosed with ALS within three quarters of their first bulbar symptom,; whereas only 21% of patients with limb-onset disease were diagnosed within three quarters of their first symptom.

Although a broad range of symptoms was included for review in this analysis, the cardinal symptoms of muscle weakness and speech difficulties (speech disturbances and voice resonance disorder) were more frequently reported in the ALS cohort in the eight quarters before diagnosis compared with the Medicare cohort (Table 2). In addition, a select number of diagnoses present in less than 10% of the ALS cohort were observed at a substantially higher rate than in the Medicare cohort (Table 3). The presence of myopathy, cervical/thoracic or lumbar spondylosis with myelopathy, or muscular disuse atrophy was associated with a longer median time to diagnosis of ALS from the onset of the first limb or bulbar symptom compared with that determined for the entire cohort. This increased time was particularly marked for patients given a diagnosis of spondylosis with myelopathy.

**Table 2:** Symptoms/diagnoses observed at a frequency > 10% in the ALS cohort* compared with the Medicare cohort^†^

**Symptom group**	**Symptom/diagnosis**	Pre-ALS frequency, %*	**Medicare frequency, %**^**†**^	**Prevalence ratio**^**‡**^
Bulbar	Speech disturbance	24	2	15
Bulbar	Voice resonance disorder	15	1	11
Limb	Muscle weakness (generalized)	30	5	5.9
Limb	Lack of coordination	13	3	4
Bulbar	Dysphagia^§^	27	7	3.9
Nerve	Hereditary and idiopathic peripheral neuropathies^§^	35	10	3.7
Nerve	Cervicalgia	18	5	3.7
Limb	Gait abnormality/difficulty walking	32	11	2.8
Other	Transient cerebral ischemia	13	5	2.8
Other	Loss of weight	17	6	2.7
Other	Stroke	11	4	2.4
Other	Lumbago/backache^§^	52	27	1.9
Other	Malaise and fatigue	56	32	1.8
Other	Syncope and collapse	26	14	1.8
Limb	Pain in limb	44	27	1.6
Other	Esophageal reflux	31	21	1.5
Other	Constipation	15	10	1.5
Respiratory	Pulmonary collapse/failure	17	12	1.4
Other	Dizziness and giddiness	12	8	1.4
Other	Swelling in limb	11	8	1.4
Respiratory	Respiratory difficulties^§^	62	49	1.3
Other	Hypothyroidism	27	22	1.3
Other	Nausea, vomiting, etc.	11	9	1.3
Limb	Pain in joint	31	26	1.2
Respiratory	Bronchitis	18	14	1.2
Respiratory	Chronic airway obstruction	19	17	1.1
Respiratory	Acute upper respiratory infection	12	11	1.1

ALS, amyotrophic lateral sclerosis.

*Frequency > 10% in the eight quarters before diagnosis of ALS (not including the quarter of diagnosis).

^†^Prevalence in the Medicare cohort.

^‡^Percentage of patients in the ALS cohort having a given symptom/the percentage of patients in the Medicare cohort with the same symptom.

^§^Includes multiple symptom types.

**Table 3:** Symptoms/diagnoses* and median time to diagnosis

**Symptom group**	**Symptom/diagnosis**	**Pre-ALS frequency, %**^**†**^	**Medicare frequency, %**^**‡**^	**Prevalence ratio**^**§**^	**Median time to diagnosis from first limb/bulbar symptom, years**^**‖**^
Nerve	Chronic inflammatory demyelinating polyneuropathy	2	< 1	30.5	ND
Nerve	Myopathy (including myopathy with weakness)	8	< 1	28.6	2.75
Nerve	Unspecified disease of spinal cord (including myelopathy NOS)	5	< 1	19.6	2.25
Nerve	Cervical/thoracic or lumbar spondylosis with myelopathy	6	1	11.2	3.50
Limb	Atrophy, muscular disuse	5	1	5.0	2.75

ALS, amyotrophic lateral sclerosis; ND, not determined because of small sample of patients with chronic inflammatory demyelinating polyneuropathy in the time to diagnosis cohort; NOS, not otherwise specified.

*Observed at a frequency < 10% with a prevalence ratio ≥ 5 compared with the Medicare cohort.

^†^In the eight quarters before ALS diagnosis, not including the quarter of diagnosis (n = 399).

^‡^Prevalence in the Medicare cohort.

^§^Percentage of patients in the ALS cohort having a given symptom/ the percentage of patients in the Medicare cohort with the same symptom.

^‖^In the time to diagnosis cohort (n = 272).

Further analysis of selected cardinal limb and bulbar symptoms identified that, for each of these symptoms, there was a proportion of patients who presented eight quarters or more before being diagnosed with ALS, particularly patients with gait abnormalities or difficulty walking (27% and 33%, respectively; Table 4). The more aggressive nature of bulbar-onset disease was reflected in the vast majority of patients that received their first diagnosis of speech disturbance, voice resonance disorder, or dysphagia 0 to three quarters before ALS was diagnosed.

**Table 4:** Average, median, and distribution by quarter of onset relative to ALS diagnosis for selected symptoms in the ALS cohort

				**Distribution of patients by time from symptom onset to ALS diagnosis (in quarters)**			
**Symptom group**	**Symptom**	**Average, quarters**	**Median, quarters**	**0–3, %**	**4–7, %**	**8–11, %**	**12+, %**
Limb	Muscle weakness	2.64	1.0	75	12	7	5
	Gait abnormality	4.53	2.0	61	12	12	15
	Difficulty in walking	5.18	3.0	52	15	21	12
	Lack of coordination	3.26	1.0	68	16	9	7
Bulbar	Speech disturbance	1.83	1.0	89	7	3	1
	Voice resonance disorder	3.00	2.0	76	8	11	5
	Dysphagia	1.07	1.0	95	5	0	0

ALS, amyotrophic lateral sclerosis.

The most common diagnostic tests employed in the eight quarters before diagnosis (not including the quarter of diagnosis) included sensory and motor nerve conduction tests, magnetic resonance imaging (MRI) and computed tomography scans, and electromyography of limbs, abdomen, or spine (Table 5). With the exception of exams of the throat and/or upper gastrointestinal tract, these tests were conducted in a higher proportion of the patients with limb-onset subgroup compared with the bulbar-onset subgroup. In the limb-onset subgroup, time to diagnosis was similar in patients with nerve conduction testing, imaging or electromyography (Table 5). In the bulbar-onset subgroup, patients who underwent an MRI of the neck and spine without dye had a longer time to diagnosis compared to the entire bulbar-onset subgroup (2.5 years and 1.25 years, respectively), whereas an MRI of the brain with or without dye was associated with a shorter time to diagnosis (0.25 years). A positive benefit on time to diagnosis was also measured for the utilization of motor nerve conduction tests in patients with bulbar-onset disease (0.25 years and 0.5 years compared with 1.25 years for the entire bulbar-onset subgroup); sensory nerve conduction tests were associated with a longer time to diagnosis (2.5 years).

**Table 5:** Diagnostic testing frequency and median time to diagnosis

**Diagnostic test**	Total ALS cohort*		**Limb-onset subgroup**		**Bulbar-onset subgroup**	
	**Percent with test**^**†**^	**Median time to diagnosis, years**^**‡**^	**Percent with test**^**†**^	**Median time to diagnosis, years**^**‡**^	**Percent with test**^**†**^	**Median time to diagnosis, years**^**‡**^
Nerve conduction testing						
Sensory nerve conduction test	38	2.75	41	2.75	30	2.50
Motor nerve conduction test with F-wave	26	2.75	30	2.75	18	0.25
Motor nerve conduction test without F-wave	23	2.75	24	2.88	18	0.50
Imaging studies						
MRI of neck and spine without dye	25	2.50	29	2.75	15	2.50
CT scans (head or brain) with/without contrast material	39	2.50	43	2.75	19	1.25
MRI of brain with/without dye	28	2.75	29	2.75	28	0.25
Electromyography						
Limb electromyography (2 extremities and related paraspinal areas)	19	2.75	22	2.50	12	ND
Limb electromyography (1 extremity and related paraspinal areas)	16	2.50	18	2.75	10	ND
Other						
Exam of throat and/or upper gastrointestinal tract	22	2.50	15	3.00	48	2.50

ALS, amyotrophic lateral sclerosis; CT, computed tomography; MRI, magnetic resonance imaging, ND, not determined, frequency of testing < 15%.

*n = 399.

^†^In the eight quarters before ALS diagnosis, not including the quarter of diagnosis.

^‡^In the time to diagnosis cohort (n = 272).

## Discussion

Claims for patients in the ALS cohort during the pre-diagnosis period encompassed a wide range of symptoms. The most frequent symptoms before diagnosis in patients in the ALS cohort were respiratory difficulties, malaise and fatigue, and lumbago/backache. While other symptoms (e.g., peripheral neuropathies, gait/walking issues, muscle weakness) occurred less frequently, rates were substantially higher in patients in the ALS cohort compared with those in the Medicare cohort. It is possible that increased physician awareness of these non-cardinal symptoms, in conjunction with more widely known classical symptoms of ALS such as dysphagia, could drive the referral of pre-diagnosis patients and/or earlier diagnosis of ALS.

Limb- and bulbar-onset ALS occurred at rates relative to one another that are consistent with those in published literature [4]. In this analysis, patients with bulbar-onset disease were diagnosed more quickly than those with limb-onset disease. Turner et al. [15] also found that diagnostic latency was shorter for patients with bulbar symptoms than those with lower limb symptoms.

Consistent with data from other populations, a delay in the diagnosis of ALS in the US Medicare population was observed [4,12]. For cardinal limb-onset symptoms such as gait abnormality and difficulty walking, a high proportion of patients exhibited these symptoms at least eight quarters before being diagnosed with ALS. The early presence of these symptoms highlights the importance of educating neurologists on these initial clinical signs and symptoms of ALS to minimize diagnostic delays. Interestingly, specific conditions (myopathy, cervical/thoracic or lumbar spondylosis with myelopathy, muscular disuse atrophy) that were relatively uncommon in the ALS population studied, but still more prevalent than in the general Medicare population, were associated with a longer time to diagnosis of ALS. This finding suggests that “incorrect” diagnoses, particularly spondylosis with myelopathy, may further delay the formal diagnosis of ALS.

The use of recommended diagnostic testing was less than would be expected given current clinical guidelines for the diagnosis of ALS [16]. The Awaji criteria recommend the incorporation of electrophysiologic data along with the assessment of clinical status [17]. In the current analysis, sensory and motor nerve conductions tests were the most frequent diagnostic tests performed before the quarter of diagnosis of ALS.

Overall, the median delay in time to diagnosis was consistent across testing modalities in limb-onset ALS. This highlights the difficulty of making the diagnosis even when recommended testing is conducted. In patients with bulbar-onset ALS, test selection influenced time to diagnosis. In this group, a positive benefit was observed for motor nerve tests, whereas sensory nerve tests were associated with a longer time to diagnosis. Specific electromyographic tests were found to be used in a minority of patients prior to the quarter of diagnosis of ALS, and their utilization was not associated with a shorter time to diagnosis in the limb-onset subgroup. In a systematic review, the incorporation of electrophysiologic testing increased the number of patients identified as having probable or possible ALS [17]. However, in the context of these results, the impact of neurophysiologic testing on time to diagnosis should be further explored.

Several limitations of this study should be noted. First, the list used for symptom identification was reviewed by only one ALS expert; use of a panel could have generated a different ranking for high and moderate likelihood symptoms. Overall, limb symptoms were less specific than bulbar symptoms. The longer time to diagnosis might have been overestimated by limb symptoms that represented unrelated medical comorbidity rather than ALS. In addition, while the Medicare database offered a large population for research purposes, it only included those patients enrolled in Medicare and did not include patients covered under commercial insurance. Medicare insurance is generally available for US citizens after their 65th birthday. Although select patient groups under the age of 65 years are eligible for Medicare (e.g., patients with ALS, patients with Social Security disability beneficiaries, patients with end stage renal disease), these groups are not representative of the average patient under the age of 65 years, and were therefore excluded from the analysis. Moreover, the small sample size limited the strength of the analyses.

## Conclusions

A diagnostic delay is evident in this analysis of the US Medicare population. Overall, the results of this exploratory analysis support the need for additional education and tools for health care providers to increase awareness of the symptoms of ALS and promote prompt referral to ALS specialists for expert diagnosis. Early recognition of ALS symptoms is key to a more efficient referral and diagnostic process for patients with ALS. Additional studies are needed to confirm the findings from this analysis.

## Abbreviations

ALS: Amyotrophic lateral sclerosis; CMS: US Centers for Medicare & Medicaid Services; CT: Computerized tomography; ICD-9-CM: International Classification of Diseases Ninth Revision, Clinical Modification; MRI: Magnetic resonance imaging; ND: Not determined; NOS: Not otherwise specified; Q1: Quarter one; Q4: Quarter four.

## Competing interests

James R. Williams and Douglas A. Kerr are employees of Biogen Idec and own stock in the company. David Fitzhenry, Lauren Grant, and Derek Martyn are employees of Trinity Partners, LLC. Initial analysis conducted by Trinity Partners was funded by Biogen Idec.

## Authors’ contributions

DF, LG, and DM conducted the analyses. DK provided guidance on data collection. All authors were involved in interpretation of results and have read and approved the final manuscript.

## Pre-publication history

The pre-publication history for this paper can be accessed here:

http://www.biomedcentral.com/1471-2377/13/160/prepub
